# FEASIBILITY OF USING SMARTPHONES AS A TOOL TO PROMOTE PHYSICAL ACTIVITY IN OLDER ADULTS

**DOI:** 10.1093/geroni/igad104.2817

**Published:** 2023-12-21

**Authors:** Josephine Elumeze, Erika Ortiz Sarmiento, Winnie Chu, Ciaran Friel, Ann Swartz, Alyson K Myers, Christian Nouryan, Edith Burns

**Affiliations:** Zucker School of Medicine Hofstra Northwell, New Hyde Park, New York, United States; Zucker School of Medicine Hofstra Northwell, New Hyde Park, New York, United States; Zucker School of Medicine Hofstra Northwell, New Hyde Park, New York, United States; Feinstein Institutes for Medical Research, Zucker School of Medicine Hofstra Northwell, Manhasset, New York, United States; University of Wisconsin-Milwaukee, Milwaukee, Wisconsin, United States; Montefiore/Einstein, Bronx, New York, United States; Northwell Health, Manhasset, New York, United States; Zucker School of Medicine at Hofstra/Northwell, Manhasset, New York, United States

## Abstract

Smartphones have potential to allow asynchronous education, individualized feedback and coaching around physical activity (PA) though it’s unclear if older adults make use of these capabilities. We conducted anonymous, semi-structured interviews/surveys on current PA levels, and experience with mobile technology and PA programs with a convenience sample of older adults, and family members/caregivers receiving care at a large geriatrics academic practice over a 3-month period. Data included demographics, experience with smartphone and PA applications. Current levels of PA were measured with the International Physical Activity Questionnaire (IPAQ). Comparisons of PA levels based on mild vs moderate vs vigorous activity. 25 completed surveys, most of whom were female (67%) and identified as non-White (68%), with a mean age of 80.8 years, range 67-96. The majority (84%) reported owning smartphones, 8% reported using PA apps, and 8% used wearable activity trackers (WATs). 44% walked at least 10 minutes per day, but only 32% reported attaining moderate PA, and 12% vigorous PA. Average time/day walking 1.1-hour, average time/day moderate activity 0.74 hours. Both younger (age≤80 years) and older (age>80 years) adults were similar in owning Smartphones (85% vs. 83%) and using WATs (8% vs. 8%). Only the younger group used PA apps (15% vs. 0%). The ubiquity of Smartphones especially among such a demographically heterogeneous group suggests this may be a viable tool for increasing PA among a wide range of older adults. Next steps should address low rates of PA app use, intergenerational differences, and the possibility of incorporating socialization with technology.

